# Synthesis of [^18^F]F-γ-T-3, a Redox-Silent γ-Tocotrienol (γ-T-3) Vitamin E Analogue for Image-Based In Vivo Studies of Vitamin E Biodistribution and Dynamics

**DOI:** 10.3390/molecules25235700

**Published:** 2020-12-03

**Authors:** Peter Roselt, Carleen Cullinane, Wayne Noonan, Hassan Elsaidi, Peter Eu, Leonard I. Wiebe

**Affiliations:** 1 The Centre for Molecular Imaging and Translational Research Laboratory, The Peter MacCallum Cancer Centre, 305 Grattan Street, Melbourne, VIC 3000, Australia; peter.roselt@petermac.org (P.R.); carleen.cullinane@petermac.org (C.C.); wayne.noonan@petermac.org (W.N.); peter.eu@petermac.org (P.E.); 2 Department of Pharmaceutical Chemistry, Faculty of Pharmacy, University of Alexandria, El Sultan Hussein St. Azarita, Alexandria 21521, Egypt; elsaidi@ualberta.ca; 3 Department of Oncology, Faculty of Medicine and Dentistry, University of Alberta, Edmonton, AB T6G 2E1, Canada; 4 Katz Group Centre for Pharmacy and Health Research, Faculty of Pharmacy and Pharmaceutical Sciences, University of Alberta, Edmonton, AB T6G 2E1, Canada

**Keywords:** nutraceuticals, antioxidants, Vitamin E, Vitamin E analogues, tocopherol (TP), tocotrienol (T-3), γ-T-3, F-γ-T-3, radiofluorination, [^18^F]F-γ-T-3, F-18 positron emission tomography (F-18 PET)

## Abstract

Vitamin E, a natural antioxidant, is of interest to scientists, health care pundits and faddists; its nutritional and biomedical attributes may be validated, anecdotal or fantasy. Vitamin E is a mixture of tocopherols (TPs) and tocotrienols (T-3s), each class having four substitutional isomers (α-, β-, γ-, δ-). Vitamin E analogues attain only low concentrations in most tissues, necessitating exacting invasive techniques for analytical research. Quantitative positron emission tomography (PET) with an F-18-labeled molecular probe would expedite access to Vitamin E’s biodistributions and pharmacokinetics via non-invasive temporal imaging. (*R*)-6-(3-[^18^F]Fluoropropoxy)-2,7,8-trimethyl-2-(4,8,12-trimethyltrideca-3,7,11-trien-1-yl)-chromane ([^18^F]F-γ-T-3) was prepared for this purpose. [^18^F]F-γ-T-3 was synthesized from γ-T-3 in two steps: (i) 1,3-di-*O*-tosylpropane was introduced at C6-O to form TsO-γ-T-3, and (ii) reaction of this tosylate with [^18^F]fluoride in DMF/K_222_. Non-radioactive F-γ-T-3 was synthesized by reaction of γ-T-3 with 3-fluoropropyl methanesulfonate. [^18^F]F-γ-T-3 biodistribution in a murine tumor model was imaged using a small-animal PET scanner. F-γ-T-3 was prepared in 61% chemical yield. [^18^F]F-γ-T-3 was synthesized in acceptable radiochemical yield (RCY 12%) with high radiochemical purity (>99% RCP) in 45 min. Preliminary F-18 PET images in mice showed upper abdominal accumulation with evidence of renal clearance, only low concentrations in the thorax (lung/heart) and head, and rapid clearance from blood. [^18^F]F-γ-T-3 shows promise as an F-18 PET tracer for detailed in vivo studies of Vitamin E. The labeling procedure provides acceptable RCY, high RCP and pertinence to all eight Vitamin E analogues.

## 1. Introduction

**Figure 1:**
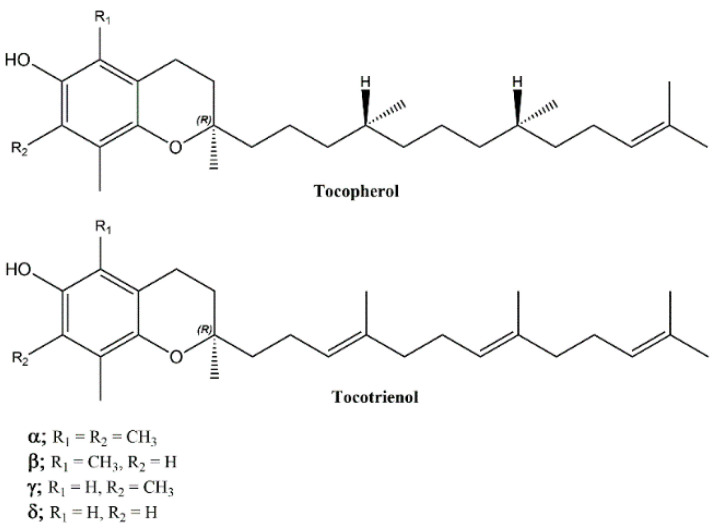
Chemical structures of the Vitamin E analogues.

**Figure 2:**
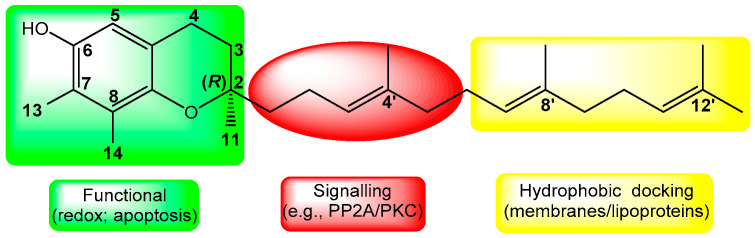
Tocotrienol (T-3) chemical structure and molecular biology of its major structural domains (adapted from [2]).

The Vitamin E family is comprised of eight chromanol analogues that fall into two subfamilies, tocopherols (TPs) and tocotrienols (T-3s). These families are characterized by the hydrocarbon chain at chromanol-C2. Whereas T-3s have a C2 isoprenoid side chain with three non-conjugated double bonds (C3′–C4′, C7′–C8′, C11′–C12′), the TPs have an identical but fully saturated chain. The four naturally occurring TP and T-3 analogues (α-, β-, γ-, δ-) differ from each other only through variations in methyl substitutions at C5, C7 and C8 on the aromatic chromanol ring (Figure 1 and Figure 2) [1].

Vitamin E analogues are important natural antioxidants that protect cells by interacting with free radicals. Importantly, there is growing evidence that other medicinal properties including anti-cancer, anti-inflammatory, and neuroprotective activity may be as important as their antioxidant action. Specific biochemical interactions may lower blood cholesterol and blood pressure, reverse atherosclerosis, minimize stroke-related brain damage, stimulate hair regrowth, and prevent sun damage to skin [2,3,4,5]. The Vitamin E analogue γ-T-3, present in many plant oils but especially palm, annatto and rice bran oils, is of particular interest to cancer researchers [6], and has been selected as the representative T-3 analogue in the current manuscript.

The pharmacology and molecular biology of Vitamin E are not fully resolved [5]. In general, the bicyclic portion (functional domain; Figure 2) is responsible for the antioxidant properties, whereas the hydrocarbon side chain at C2 has two functions: the proximal portion imparts signaling activity, and the distal (terminal) hydrocarbon tail imparts additional hydrophobicity which facilitates interaction with lipophilic cell components [2]. The side-chain terminus is subject to oxidative attack by cytochrome P450 4F2 (CYP4F2) at C-13′, leading to ω-hydroxylation followed by a cascade of five beta oxidations starting with C-13′. This truncation of the side chain ends with the water-soluble carboxyethylhydroxychroman or carboxymethylbutylhydroxychroman cores [7,8,9]. Metabolism of the aromatic ring of the chroman moiety has been reported to give rise to minor metabolites [10].

Gamma tocotrienol (γ-T-3), the analogue of interest because of its anti-cancer effects, has methyl substituents at C7 and C8 (Figure 1) and acts at several molecular foci [11]. The scientific literature addressing Vitamin E is too extensive to review in this paper; interested readers are referred to the many contemporary reviews of its dietary and medicinal attributes [5,12,13,14].

The disposition of T-3′s within body tissues and association with various membranes are reported to be superior to those of other members of the natural Vitamin E group. The low bioavailability of all Vitamin E analogues remains a major concern for harnessing their medicinal properties. For example, the bioavailability of γ-T-3 has been reported to be ~10% following a single oral bolus dose of 10 mg/kg to rats [15].

**Scheme 1:**
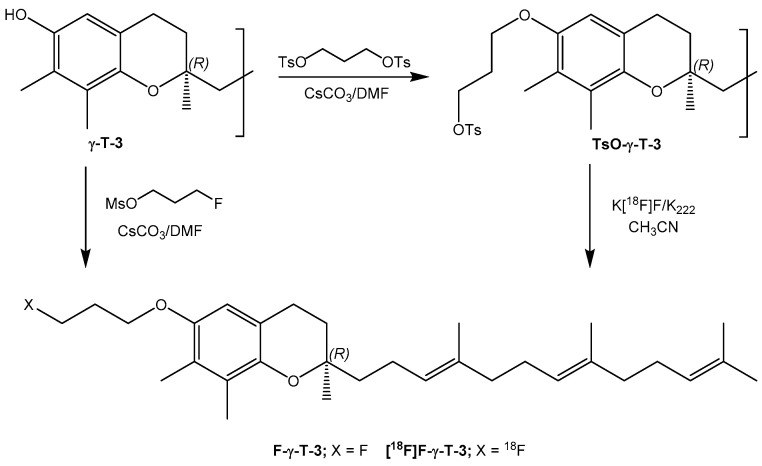
Roadmap for the synthesis of F-γ-T-3 and [^18^F]F-γ-T-3. Reagents and conditions for F-γ-T-3: MsF, Et_3_N, DCM, r.t., 30 min, (92.7% yield), followed by CsCO_3_, DMF, r.t., overnight, (61% yield); for TsO-γ-T-3, CsCO_3_, DMF, r.t., overnight (48% yield); and for radiosynthesis of [^18^F]F-γ-T-3, K [^18^F]F, K_222_, CH_3_CN, 100 °C, 10 min (12% RCY, >99% RCP).

The syntheses (Scheme 1) of F-γ-T-3 and [^18^F]F-γ-T-3 are now reported, together with a PET image of the biodistribution of [^18^F]F-γ-T-3 following i.v. injection into a murine tumor model. The objective of this program is to develop reagents that will facilitate administration, bioavailability, clearance, and distribution studies of Vitamin E dynamics in animal models and humans through the application of imaging techniques [16,17].

## 2. Results

### 2.1. Chemistry

**Figure 3:**
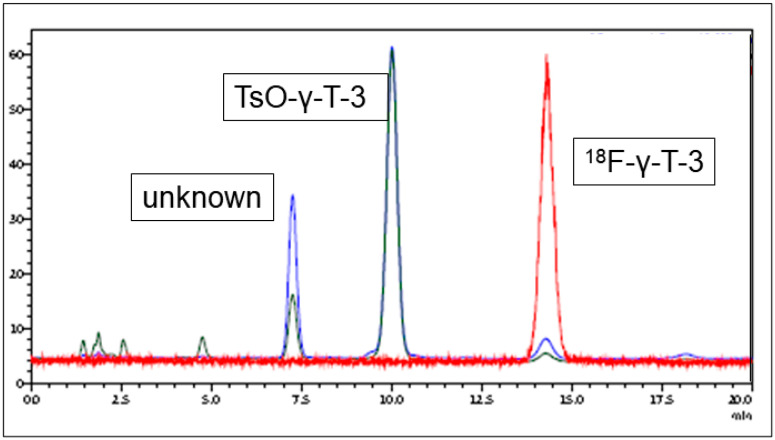
A representative HPLC radio-uv co-chromatogram of the reaction mixture after radiofluorination. The 291 and 254 nm peaks eluting at 14.4 min, and the radioactive peak (14.4 min; red trace) represent the co-mixture of authentic F-γ-T-3 (small blue peaks under the radioactive peak) and product [^18^F]F-γ-T-3 in the reaction mixture. Absorption peaks in the blue trace (291 nm) correspond to the major UV absorption band of the methyl-substituted 6-chromanol (benzopyran) ring system (e.g., F-γ-T-3, TsO-γ-T-3), and the dark green trace (254 nm) is a less selective indicator of aryl compounds. The main UV absorption peaks eluting at 10.1 min (291 and 254 nm) represent unconsumed TsO-γ-T-3; the absorption peaks eluting at earlier times are unidentified. The isocratic reverse phase HPLC system is described in the text.

F-γ-T-3 and TsO-γ-T-3 were synthesized from γ-T-3 in acceptable chemical yields of 61% and 48%, respectively (Scheme 1). The isocratic HPLC system developed for this work provided good separation between TsO-γ-T-3 and F-γ-T-3. A chromatogram of a mixture of TsO-γ-T-3 and F-γ-T-3 showed the presence of several minor impurities which were not identified (Figure 3). Although no quantitative stability studies were undertaken, standard solutions of TsO-γ-T-3 and F-γ-T-3 in ethanol showed minimal chemical change over a period of four months when stored at room temperature unprotected from light. Solutions of TsO-γ-T-3 in propylene glycol were less stable, appearing to break down within a few hours. More work would be required to establish the stability of TsO-γ-T-3 and F-γ-T-3 under other storage conditions.

### 2.2. Radiofluorination

Radiofluorination of TsO-γ-T-3 provided adequate radiochemical yield (RCY 12%) without further optimization (Scheme 1). HPLC purification afforded 0.5–1 GBq high-purity (RCP > 99%) [^18^F]F-γ-T-3. The gradient HPLC method produced high radiochromatographic purity product with no detectable radiochemical impurities. As expected, the high-specific-activity product obtained via nucleophilic radiofluorination was not detectable by UV absorption, but addition of internal standard F-γ-T-3 produced a peak at 14.4 min, directly underneath the radioactive peak of [^18^F]F-γ-T-3 upon co-chromatography (Figure 3). The other UV-active peaks were not identified, but a larger peak at approximately 7.2 min showed UV absorbance at both 254 and 291 nm, and could therefore be the Tos-γ-T-3 hydrolysis product formed during work up of the radiofluorination reaction (Figure 3). The calculated partition coefficients (log*P*) of F-γ-T-3 and γ-T-3 were 9.07 and 8.80, respectively.

### 2.3. PET Imaging

**Figure 4:**
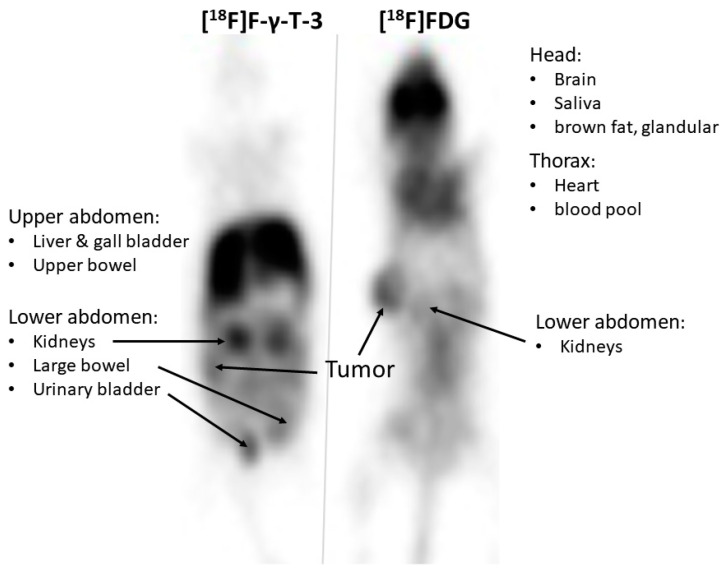
F-18 PET images showing the biodistribution of radioactivity in a mouse following i.v. tail veil injections of [^18^F]F-γ-T-3 (left image) and reference [^18^F]FDG (right image). The [^18^F]F-γ-T-3 image was captured 2 h after injection, and the [^18^F]FDG was obtained 90 min post-injection, using the same animal 24 h following the [^18^F]F-γ-T-3 study.

PET images of mice after i.v. injection of [^18^F]F-γ-T-3 were acquired sequentially at 1, 2 and 3 h post-injection. For biodistribution reference, [^18^F]FDG images were acquired in the same animals 90 min post-injection of [^18^F]FDG, 24 h after their [^18^F]F-γ-T-3 dose. A representative 2 h [^18^F]F-γ-T-3 image shows no appreciable retention of radioactivity in lung or blood pool, but substantial uptake in the upper abdominal (hepato-biliary-small intestine) region, moderate renal/urinary and lower GI radioactivity clearance, and visible uptake in the implanted MDA-MB-231 tumor (Figure 4).

## 3. Discussion

### 3.1. Radiolabeling

There are very few options for the introduction of elemental radiolabels into Vitamin E analogues based on their chemical structure and ascribed regional structure-activity relationships (Figure 1 and Figure 2). The radiolabeling options fall into two main categories: those that impact the redox/bio-oxidation properties (e.g., alteration/substitution at C6-OH; redox silencing), and those that alter signaling, docking and metabolic degradation ascribed to the C2 chain (e.g., substitution on isoprenoid double bonds of T-3s). The latter option requires creation of a reactive centre somewhere along the C2 chain, a challenging chemical synthesis, and creates substantial alterations in the biophysical properties of these molecules. Incorporation of tracer elements at aromatic sites other than C6-O would similarly necessitate extensive chemical modification. In short, chemical alteration at either the side chain or the aromatic moiety will temper interaction with membranes and macromolecular targets, alter redox characteristics and/or modulate absorption and biodistribution patterns. The search for more active synthetic derivatives of natural TPs and T-3s focus primarily on substitution at C6-O [18,19,20,21]. Introduction of F-18 at C6-O via a prosthetic linking arm seems to be the best option for radiofluorination of all Vitamin E analogues.

Two options are available for radiofluorination at C6-O. The approach based on electrophilic radiofluorination with [^18^F]F_2_ has several drawbacks including technical challenges of working with electrophilic fluorine, lower specific activity of the final labeled products, and low regioselectivity of substitution in aromatic systems [22]. The latter constraint could be addressed using organometallic precursors, but the synthesis of these precursors presents its own special challenges [23]. The second radiolabeling option is nucleophilic radiofluorination of an appropriate alkyl linking group introduced as a substituent on C6-O. The relative ease of nucleophilic substitution, the high specific activities available, and controlled regiospecificity all mitigate in its favor [24]. Electrophilic and nucleophilic radiofluorination at C6-O both give rise to new compounds that are redox silent, and although this affects the antioxidant properties, this has not been considered to be a major impediment to the development of more active derivatives [20].

The catabolism of Vitamin E analogues occurs via sequential oxidations, first by ω-oxidation of a terminal methyl group of the isoprenoid side chain, followed by sequential β-oxidations to the corresponding carboxyethyl hydroxychromans (CEHCs) and eventual urinary excretion in urine as glucuronated or sulfated forms [8,25]. Although qualitative metabolic effects have not been demonstrated as a result of aromatic ring substitution patterns and substituents, quantitative effects (rates) have been reported and ascribed to differences in binding to tocopherol-ω-hydroxylase (CYP4F2), the enzyme responsible for ω-hydroxylation [26].

The current demonstration is based on the nucleophilic radiofluorination approach using bifunctional fluoroalkanes, e.g., ^18^F(CH_2_)_n_X (*n* = 1–3, X = Br, OMes, OTos) [27]. This approach (Scheme 1) required the syntheses of reference F-γ-T-3 and the radiofluorination precursor TsO-γ-T-3, both of which proceeded in a straight-forward manner. As depicted in Scheme 1, mesylation of 3-fluoro-1-propanol afforded intermediate **2** which was subjected to nucleophilic substitution of its mesyl group with γ-T-3 to furnish the target F-γ-T-3 in 61% yield. The tosyl-radiofluorination precursor, TsO-γ-T-3, was prepared in a 47% yield via nucleophilic displacement of one tosylate group of the di-tosylate **3** with γ-T-3. Standard work up of these compounds required attention to their instability under silica gel chromatography and sensitivity to UV light.

**Figure 5:**
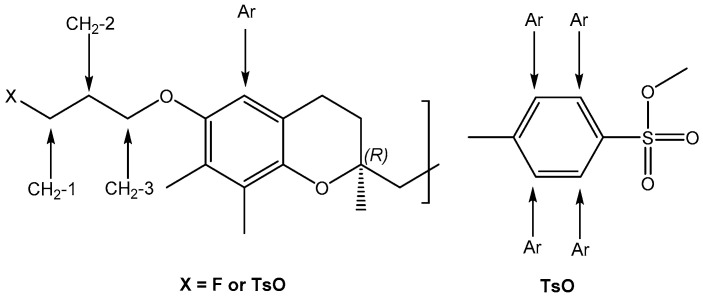
NMR numbering of target compounds F-γ-T-3 and [^18^F]F-γ-T-3.

NMR analyses of the non-radioactive compounds focused on resonance shifts arising from substitution of the C6 hydroxyl group of the aromatic ring by ether-linked propyl groups (3-tosylpropyl- and 3-fluoropropyl-). Assignment of proton and carbon resonances of all natural Vitamin E analogues remains incomplete despite the application of multidimensional NMR (HSQC, COSY, ADEQUATE(1,1), C-H HMBC, and NOESY) techniques [28]. Resonances introduced by the 3-(F/TsO)-propyloxy substituent at C6, replacing hydrogen on the C6 hydroxy of γ-T-3, are reported in the experimental section as CH_2_-1, CH_2_-2 and CH_2_-3 (Figure 5).

**Figure 6:**
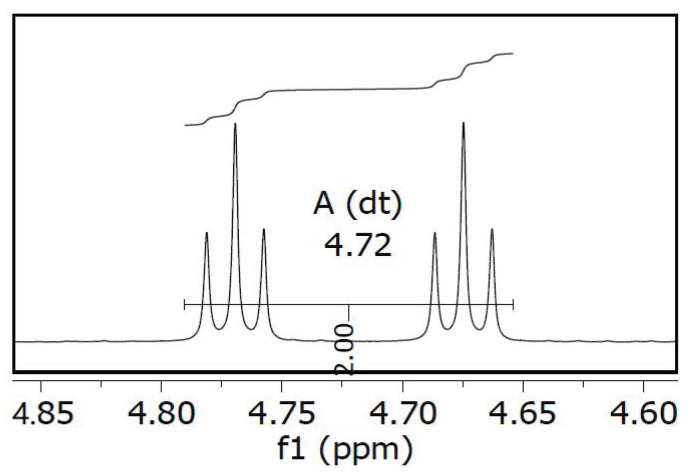
The partial ^1^H-NMR spectrum of F-γ-T-3 reveals the distinctive coupling pattern of the fluoropropyloxy substituent at C6-O. These resonances are well separated from other aliphatic-H resonances and are therefore useful in distinguishing F-γ-T-3 from non-substituted T-3s.

Figure 6 illustrates the resonances of these CH_2_(s), showing the distinctive coupling pattern indicative of the presence of the propyloxy side arm, especially CH_2_-1 of F-γ-T-3 which showed the distinctive coupling pattern of dt (doublet of triplet) with coupling constants *J* = 47.1 and 5.9 Hz, indicating F-H and H-H coupling, respectively (Figure 6).

The preparation of radiofluorination precursors in which alkyl tosylates are appended to the core molecule is a technique frequently used to radiofluorinate aryl cores. In such reactions, propyl tosylates may be preferred for reasons of stability and radiochemical yield. It has been reported that in the radiofluorination of the propyl tosylate or ethyl tosylate carbocyclic nucleoside precursors, the respective radiochemical yields were 10% and 4–5%, respectively [29]. Another approach to radiofluorinate Vitamin E analogues at C6-O would be to first prepare [^18^F]fluoroalkyl tosylate ([^18^F]FET), and then couple this to the target phenolic hydroxyl to prepare the final compound. For example, in the radiosynthesis of l-5-(2-[^18^F]fluoroethoxy)-tryptophan (5-[^18^F]FETP) by [^18^F]fluoroethylation of the 5-hydroxy-l-tryptophan disodium salt, [^18^F]FET was formed in 45–60% RCY, and after sequential [^18^F]fluoroethylation of 5-hydroxy-l-tryptophan in DMSO and HPLC purification, 5-[^18^F]FETP was isolated within 65 min in 12–16% overall RCY [30].

In summary, tosyl displacement by [^18^F]fluoride on the C6-O side chain and coupling the [^18^F]fluoroalkyl moiety both produce the same radiofluorinated product, and the RCYs of both approaches compare favorably. The 12% RCY now reported for radiofluorination of TsO-γ-T-3 was not optimized, but this RCY was adequate for the subsequent animal imaging demonstration (Figure 4).

[^18^F]F-γ-T-3 was used at no-carrier-added (NCA) specific activity (SA). The theoretical SA of NCA [^18^F]fluoride is 6.3 TBq/μmol, but in reality, unintentional addition of fluorine through the ubiquitous presence of fluorine in reagents and materials reduces this to 30–150 GBq (1–5 Ci)/μmol of NCA product, although SA’s of 0.1–1.9 TBq (3–51 Ci)/μmol have been reported [31,32]. At 30–150 GBq/μmol, an injected radioactivity dose of 14.8 MBq would represent approximately 0.48 to 0.1 nmol of F-γ-T-3 (end of synthesis), an amount not likely to modulate most Vitamin E-related processes and in line with the low Vitamin E concentrations found in tissues. Pharmacokinetic parameters of Vitamin E analogues, in humans at least, do not appear to be dose dependent over a large dose range [33], keeping in mind that studies using non-labeled analogues are normally undertaken at comparatively high doses.

### 3.2. PET Imaging

PET imaging was limited to a demonstration study using two tumor-bearing mice. One hour after injection, there was no thoracic retention of the [^18^F]F-γ-T-3 tracer, whereas retention in the upper abdominal region including liver, gall bladder and upper intestine was prominent at 1 h and persisted through to 3 h post-tracer injection. Although formulation of highly lipophilic drugs for i.v. administration can be difficult, this challenge is markedly reduced for high SA radiopharmaceuticals. None the less, radiopharmaceutical preparation and formulation problems, and radiopharmaceutical administration techniques, are among the several important factors affecting the biodistribution of radiopharmaceuticals [34]. PPG is an inexpensive, non-toxic, and well-tolerated cosolvent [34] that has been used in a range of formulations as a co-solvent [35].

Both radiotracers depict tumor and blood pool, but [^18^F]FDG concentrations visually appear to be higher in the tumor and in blood. The elevated uptake of radioactivity in the thorax and head following [^18^F]FDG injection reflects high glucose utilization by heart and brain. In contrast, high uptake of radioactivity in the abdominal region following [^18^F]F-γ-T-3 injection (Figure 4) is likely to be indicative of extensive first-pass accumulation of this large (MW 624) lipophilic molecule.

Published lipophilicity estimates (log*P*) for Vitamin E analogues are inconsistent. For example, α-tocopherol (α-TP), log*Ps* range from an experimental 12.2 [14] to calculated log*P*s of 10.7 [36] and 9.04 [37]. Clearly these molecules are extremely lipophilic, and given calculated log*Ps* of 9.04 for γ-T-3 [37] and 10.08 for α-TP-acetate [38], minor substitutions and derivatizations on the chromanol moiety are unlikely to shift the log*P* beyond the range of values currently available through experimentation or calculation. The calculated F-γ-T-3 log*P* (8.95) was only marginally greater than the calculated log*P* of γ-T-3 (8.80).

The cancer cell line ATCC^®^ HTB-26™, aka MDA-MB-231, a human triple-negative epithelial mammary gland/breast adenocarcinoma initially derived from a metastatic site pleural effusion, was selected for this work because it serves as a versatile tool for many cancer-related research studies. Examples include comparative biodistributions of diverse radiotracers and studies of cell response to various agents [39,40]. Uptake of [^18^F]F-γ-T-3 by the MDA-MB-231 tumor was sufficient to visualize the tumor, but quantification of the image radioactivity disposition was not attempted. Rapid clearance of radioactivity from the blood stream was visually apparent, and with concurrent hepatic uptake, would be consistent with first-pass clearance and in line with previously reported extensive reticuloendothelial (RES) clearance and short plasma half life (~2.5–3 h) following i.v. intravenous delivery to rats [15]. However, uptake by tumor is visible in the PET image, indicating that an appreciable fraction of the injected radioactivity was not subject to first-pass clearance from blood even given the very small i.v. dose of [^18^F]F-γ-T-3.

Same-animal imaging with [^18^F]FDG 24 h after [^18^F]F-γ-T-3 injection produced expected FDG-biodistribution images. Differences in biodistributions of the two tracers included the high head, thorax and tumor uptake of [^18^F]FDG with lingering blood pool radioactivity. This biodistribution was in stark contrast to the virtual absence of uptake in head and thorax and the low levels of radioactivity in tumor and blood after [^18^F]F-γ-T-3 injection and apparent rapid clearance from blood. Emulsification of the highly lipophilic [^18^F]F-γ-T-3 in EtOH:PPG:saline (1:1:2) upon injection into the blood stream and consequent skewing of the biodistribution of [^18^F]F-γ-T-3 was considered unlikely because the chemical load of [^18^F]F-γ-T-3 was estimated to be less than 1 nmol. Importantly, the PET image provides no evidence of extravasation or a depot effect at or downstream from the injection site, and the animals displayed no acute toxicity/side effects over the 24 h period between the [^18^F]F-γ-T-3 and [^18^F]FDG injections. These observations confirm that dose formulation and injection technique were not contributing factors to the observed biodistribution of either [^18^F]F-γ-T-3 or [^18^F]FDG.

The PET image (Figure 4) represents the first ‘big picture’ of the whole-body distribution of any member of the Vitamin E family and specifically of the T-3 subfamily. This image confirms rapid blood clearance and strong hepato-biliary-intestinal accumulation of [^18^F]F-γ-T-3 after i.v. injection, which although not quantitative, augments biodistribution data for δ-T-3 from an oral dosing study [41]. The traditional use of Vitamin E analogues (as mixtures or as pure substitutional isomers) in nutraceuticals and dietary supplements underlines the importance of natural source constituents as ‘co-factors’ in their bioavailability and adds another level of complexity to the interpretation of dynamic data such as metabolism and bioavailability [26,42].

## 4. Materials and Methods

### 4.1. General

Solvents for reactions were purified by successive passage through columns of alumina and copper under an argon atmosphere. Reagents were purchased from commercial sources and used without further purification unless noted otherwise. All reactions were carried out under a positive-pressure argon atmosphere and monitored by thin-layer chromatography (TLC) on Whatman MK6F silica gel micro TLC plates (25 μm thickness) or Silica Gel G-25 UV254 (0.25 mm) microplates using hexanes:EtOAc (1:3, *v*/*v*) (solvent system A) and hexanes:EtOAc (1:1, *v*/*v*) (solvent system B) as developing solvents. TLC spots were detected under UV light and/or by charring with a solution of anisaldehyde in ethanol, acetic acid and H_2_SO_4_. Column chromatography was carried out on Merck 7734 silica gel (100–200 μm particle size).

^1^H- and ^13^C-NMR spectra (see Supplementary Materials) were recorded at 498.118 and 125.266 MHz, respectively, and ^19^F-NMR spectra were recorded at 468.652 MHz. ^1^H/^19^F-NMR chemical shifts are referenced to TMS (0.0, CDCl_3_) and ^13^C-NMR chemical shifts are referenced to CDCl_3_ (d 77.23). ^1^H-NMR data are reported as though they are first order and peak assignments are based on 2D-NMR (^1^H–^1^H COSY and HMQC) experiments (Figure 6). Mass spectra were recorded on either an Agilent 1100 LC/MS using an Agilent Zorbax C-18 column (2.1 × 50 mm, 5 μM, Agilent Technologies Inc., Mississauga, ON, CA) or Q Exactive™ Hybrid Quadrupole-Orbitrap™ Mass Spectrometer with Xcalibur Data Acquisition and Interpretation Software.

#### 4.1.1. Fluoropropyl Methanesulfonate (**2**, Scheme 1)

Et_3_N (7 mL, 51.2 mmol) was added to a solution of 3-fluoro-1-propanol (**1**; 2 g, 25.6 mmol) in DCM (30 mL) at 0 °C followed by the dropwise addition of MsCl (3 mL, 38.4 mmol). The resulting mixture was stirred at r.t. for 0.5 h. The mixture was diluted with DCM (50 mL) and washed sequentially with sat. NaHCO_3_ (2 × 50 mL), H_2_O (50 mL) and brine (50 mL). The organic solution was dried over anhydrous Na_2_SO_4_ and filtered. The filtrate was concentrated to afford 3-mesyl-1-fluoropropane, **2**, as a brown oil in 92.7% yield (3.70 g). Compound **2** was used in the subsequent step without further purification.

#### 4.1.2. (*R*)-6-(3-Fluoropropoxy)-2,7,8-trimethyl-2-(4,8,12-trimethyltrideca-3,7,11-trien-1-yl)-chromane (F-γ-T-3, Scheme 1)

Powdered Cs_2_CO_3_ (3.18 g, 9.77 mmol) was added to a mixture of **2** (1.52 g, 9.77 mmol) and γ-T-3 (1.50 g, 3.66 mmol) in DMF (15 mL). The resulting mixture was stirred at r.t. overnight, and then diluted with Et_2_O (100 mL) and washed with H_2_O (50 mL). The aqueous solution was extracted with Et_2_O (2 × 50 mL) and the resulting organic solution was washed with brine (50 mL), dried over anhydrous Na_2_SO_4_ and filtered. The filtrate was concentrated and purified by silica gel column chromatography eluted with 0–5% EtOAc in hexane to afford F-γ-T-3 as a yellowish oil (1.05 g, 61%): ^19^F-NMR (468.652 MHz, CDCl_3_): δ = −11.71 (tt, *J* = 47.1, 25.5 Hz); ^1^H-NMR (498.118 MHz, CDCl_3_) δ = 6.50 (s, 1H, Ar), 5.27–5.12 (m, 3H), 4.72 (dt, *J* = 47.1, 5.9 Hz, 2H, CH_2_–1), 4.06 (t, *J* = 6.0 Hz, 2H, CH_2_–3), 2.85–2.72 (m, 2H), 2.28–2.17 (m, 10H, including CH_2_–2), 2.17–2.10 (m, 4H), 2.05 (dd, *J* = 8.6, 4.9 Hz, 4H), 1.83 (ddt, *J* = 36.5, 13.2, 6.8 Hz, 2H), 1.75 (d, *J* = 1.4 Hz, 3H), 1.71 (dd, *J* = 9.1, 7.6 Hz, 1H), 1.69–1.64 (m, 9H), 1.64–1.59 (m, 1H), 1.33 (s, 3H); ^13^C-NMR (125.266 MHz, CDCl_3_) δ = 149.82, 145.95, 135.07, 134.95, 131.21, 125.98, 124.62, 124.26, 117.51, 109.97, 81.74, 80.43, 77.33, 77.08, 76.82, 75.27, 64.57, 64.52, 39.89, 39.77, 39.75, 31.53, 30.92, 30.76, 26.82, 26.66, 25.73, 24.06, 22.66, 22.28, 17.72, 16.04, 15.93, 11.91, 11.89; HRMS (ESI): *m*/*z* calcd. for C_31_H_48_FO_2_: 471.3638 [M + H]^+^; found: 471.3630.

#### 4.1.3. (*R*)-6-(3-Tosyl-propoxy)-2,7,8-trimethyl-2-(4,8,12-trimethyl-trideca-3,7,11-trien-1-yl)-chroman (TsO-γ-T-3, Scheme 1)

Powdered Cs_2_CO_3_ (4.24 g, 13.0 mmol) was added to a mixture of γ-T-3 (2 g, 4.87 mmol) and **3** (5 g, 13.0 mmol) in DMF (20 mL). The resulting mixture was stirred at r.t. overnight. The mixture was then diluted with EtOAc (20 mL) and water (50 mL) and finally extracted with EtOAc (2 × 20 mL). The combined organic solution was washed with H_2_O (50 mL). The organic solution was dried over Na_2_SO_4_. After filtration, the filtrate was concentrated and purified by silica gel column chromatography eluted with DCM to afford TsO-γ-T-3 as a yellow oil in 48% yield (1.45 g): ^1^H-NMR (498.118 MHz, CDCl_3_): δ = 7.78 (d, *J* = 8.2 Hz, 2H, Ar), 7.27 (d, *J* = 7.8 Hz, 2H, Ar), 6.34 (s, 1H, Ar), 5.40–4.97 (m, 2H), 4.28 (t, *J* = 6.1 Hz, 2H, CH_2_–1), 3.89 (t, *J* = 5.8 Hz, 2H, CH_2_–3), 2.78–2.66 (m, 2H), 2.41 (s, 3H, CH_3_), 2.20–2.07 (m, 6H, including CH_2_–2), 2.02–1.94 (m, 5H), 1.87–1.49 (m, 18H), 1.35–1.17 (m, 6H), 1.09–0.83 (m, 3H); ^13^C-NMR (125.266 MHz, CDCl_3_) δ = 182.23, 149.44, 145.89, 144.65, 132.92, 129.76, 127.85, 125.84, 124.30, 117.42, 109.56, 77.31, 77.06, 76.80, 75.27, 71.42, 67.48, 63.88, 39.88, 39.72, 36.93, 35.00, 31.48, 29.22, 25.71, 24.07, 23.09, 22.63, 22.23, 21.59, 17.70, 16.55, 15.82, 11.88, 11.74; HRMS (ESI): *m*/*z* calcd. for C_38_H_54_O_5_S: 623.3765 [M + H]^+^; found: 623.3760.

### 4.2. Radiosynthesis of [^18^F]F-γ-T-3 (Scheme 1)

#### 4.2.1. General Radiochemistry

Acetonitrile (CH_3_CN) and Kryptofix2.2.2 (K_222_) were obtained from Merck (Darmstadt, Germany), and dry dimethyl sulfoxide (DMSO) was purchased from Sigma Aldrich (St. Louis, MO, USA). Sep-Pak light, Accell Plus QMA and Alumina N cartridges were from Waters, Milford, MA, USA. Phenomenex Luna pre-column (C18/2, 50 × 10 mm; 5 µm), Phenomenex Nucleosil columns (C18, 250 × 10 mm; 5 µm and C18, 250 × 4.6 mm) and 0.22 µm Millex GS and LX filters were from Millipore, Billerica, MA, USA. NCA [^18^F]fluoride was obtained from a PETtrace 16.5 MeV cyclotron incorporating a high-pressure niobium target (Cyclotek(AUST) Pty. Ltd., Victoria, Australia)) via the ^18^O(p,n)^18^F nuclear reaction. F-18 separation cartridges (Waters Accell Plus QMA Sep-Pak Light, Kent, UK) were pre-conditioned with 0.5 M K_2_CO_3_ and subsequently rinsed with water. Radio-HPLC analyses were performed using a Shimadzu HPLC (SCL-10AVP system controller, SIL-10ADVP auto injector, LC-10ATVP solvent delivery unit, CV-10AL control valve, DGU-14A degasser, and SPD-10AVPV detector) Q6 coupled to a scintillation detector (Ortec 276 Photomultiplier Base with Preamplifier, Ortec 925-SCINT ACE mate Preamplifier, Amplifier, BIAS supply and SCA, and a Bicron 1M 11/2 Photomultiplier Tube).

#### 4.2.2. (*R*)-6-(3-[^18^F]Fluoropropoxy)-2,7,8-trimethyl-2-(4,8,12-trimethyltrideca-3,7,11-trien-1-yl)-chromane ([^18^F]F-γ-T-3, Scheme 1)

[^18^F]Fluoride in H_2_[^18^O]O was transferred to the Tracerlab FX_FN_ radiosynthesis module and passed through a pre-conditioned QMA cartridge. Trapped [^18^F]fluoride (3–7 GBq) was eluted to the reactor with a solution consisting of K_2_C_2_O_4_ (2.5 mg), K_222_ (10 mg) and K_2_CO_3_ (10 mL of 5 mg/mL solution) in CH_3_CN and H_2_O (1 mL, 80:20). This solution was evaporated to dryness at 65 °C under helium flow and vacuum for 7 min followed by heating at 120 °C under vacuum for a further 7 min. Tosylate precursor (TsO-γ-T-3; 10 mg) in CH_3_CN was added to the anhydrous K[^18^F]F/K_222_ residue, followed by heating at 100 °C for 10 min. The radioactive reaction mixture was then diluted with mobile phase (EtOH-H_2_O, 1.5 mL) and transferred to the loop injection vial. The reaction vial was washed further with mobile phase (1.5 mL) and transferred to the loop injection vial. Preparative HPLC [(Nucleosil C18, 300 mm × 16 mm), mobile phase H_2_O:EtOH (90:10), flow rate 3 mL/min] afforded [^18^F]F-γ-T-3 (0.5–1.0 GBq; 12% RCY; >99% RCP), in a total preparation time of 45 min. [^18^F]F-γ-T-3 was formulated in EtOH:propylene glycol (PPG):saline(0.9%) (25:25:50) and used directly for small-animal imaging studies.

### 4.3. Partition Coefficients (LogP)

Log*P*s were calculated using the *Interactive logP calculator* (Molinspiration Cheminformatics, Nova ulica, SK-900 26 Slovensky Grob, Slovak Republic). https://www.molinspiration.com/cgi-bin/properties.

### 4.4. Animal Imaging by Positron Emission Tomography (PET)

Female Balb/c nude mice (age 9 weeks) (Animal Resources Centre, Western Australia) were inoculated subcutaneously on the flank with 4 × 10^6^ MDA-MB-231 (Human Caucasian breast adenocarcinoma; ATCC^®^ HTB-26™) cells in phosphate-buffered saline (PBS)-Matrigel (1:1). Mice were weighed and tumors measured three times weekly using electronic calipers. Tumor volume (mm^3^) was calculated as length (mm)/2 × width (mm)^2^. Nineteen days later, mice with tumors (~70–380 mm^3^) were injected with [^18^F]F-γ-T-3 (14.8 MBq) in formulation solvent (140 μL). At 1 h post-dosing, the mice were anesthetised using isoflurane and imaged on a Philips Mosaic small-animal PET scanner over 10 min. The animals were returned to their housing box to recover before being re-anesthetised and imaged again at 2 h post-dose. The recovery-re-anesthetization was repeated for 3 h post-dose imaging. The following day the mice were fasted for 3 h before being injected with 14.8 MBq [^18^F]FDG and then imaged under isoflurane anaesthesia 90 min post-injection.

All procedures using animals were approved by the Peter MacCallum Cancer Centre Animal Experimentation Ethics Committee (Permit E475) and were performed in accordance with the Australian Code of Practice for the Care and Use of Animals for Scientific Purposes (ISBN 1864965975; https://www.nhmrc.gov.au/about-us/publications/australian-code-care-and-use-animals-scientific-purposes).

## 5. Conclusions

Syntheses of the reference (F-γ-T-3) and labeling precursor (TsO-γ-T-3) compounds were facile, and radiofluorination of TsO-γ-T-3 provided [^18^F]F-γ-T-3 in acceptable radiochemical yield and high radiochemical purity. Formulation of the highly lipophilic [^18^F]F-γ-T-3 in EtOH:PPG:saline (1:1:2) was well tolerated by mice following i.v. injection. Initial PET images after administration of [^18^F]F-γ-T-3 showed evidence of first-pass hepatobiliary uptake, with rapid blood clearance and no uptake in brain, heart or lungs. [^18^F]F-γ-T-3 is considered to be a useful tracer in pharmacokinetic studies of F-γ-T-3, and a highly sensitive marker for dynamic studies of Vitamin E-containing products and formulations.

## Supplementary Materials

The ^1^H-, ^13^C- and ^19^F-NMR spectra are available in Supplementary Materials.
