# Supplementary material for: Synthesis of [18F]F-γ-T-3, a Redox-Silent γ-Tocotrienol (γ-T-3) Vitamin E Analogue for Image-Based In Vivo Studies of Vitamin E Biodistribution and Dynamics
Source: Molecules. 2020 Dec 3;25(23):5700. doi: 10.3390/molecules25235700 (PMC7730577; doi:10.3390/molecules25235700)
Supplement: Supplementary file 1 [file molecules-25-05700-s001.pdf]

2018.03.28.i5_TCS063002_12.04_H1_1D
TCS063002
498.118 MHz H1 1D in cdcl3 (ref. to CDCl3 @ 7.26 ppm)
temp 26.9 C -> actual temp = 27.0 C, autoxdb probe

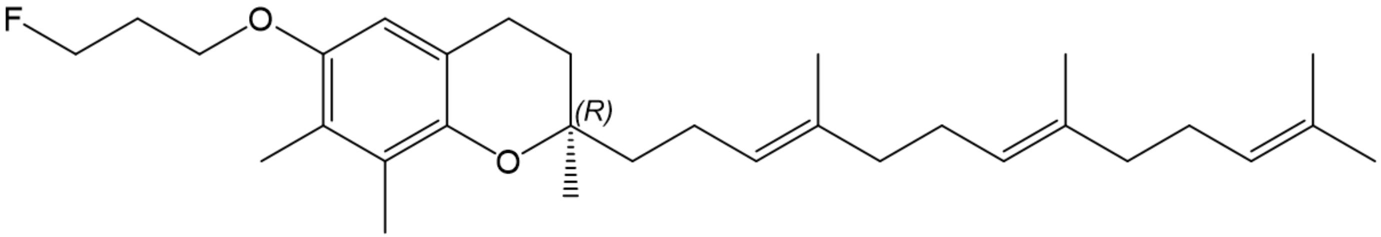

F-γ-TOCO

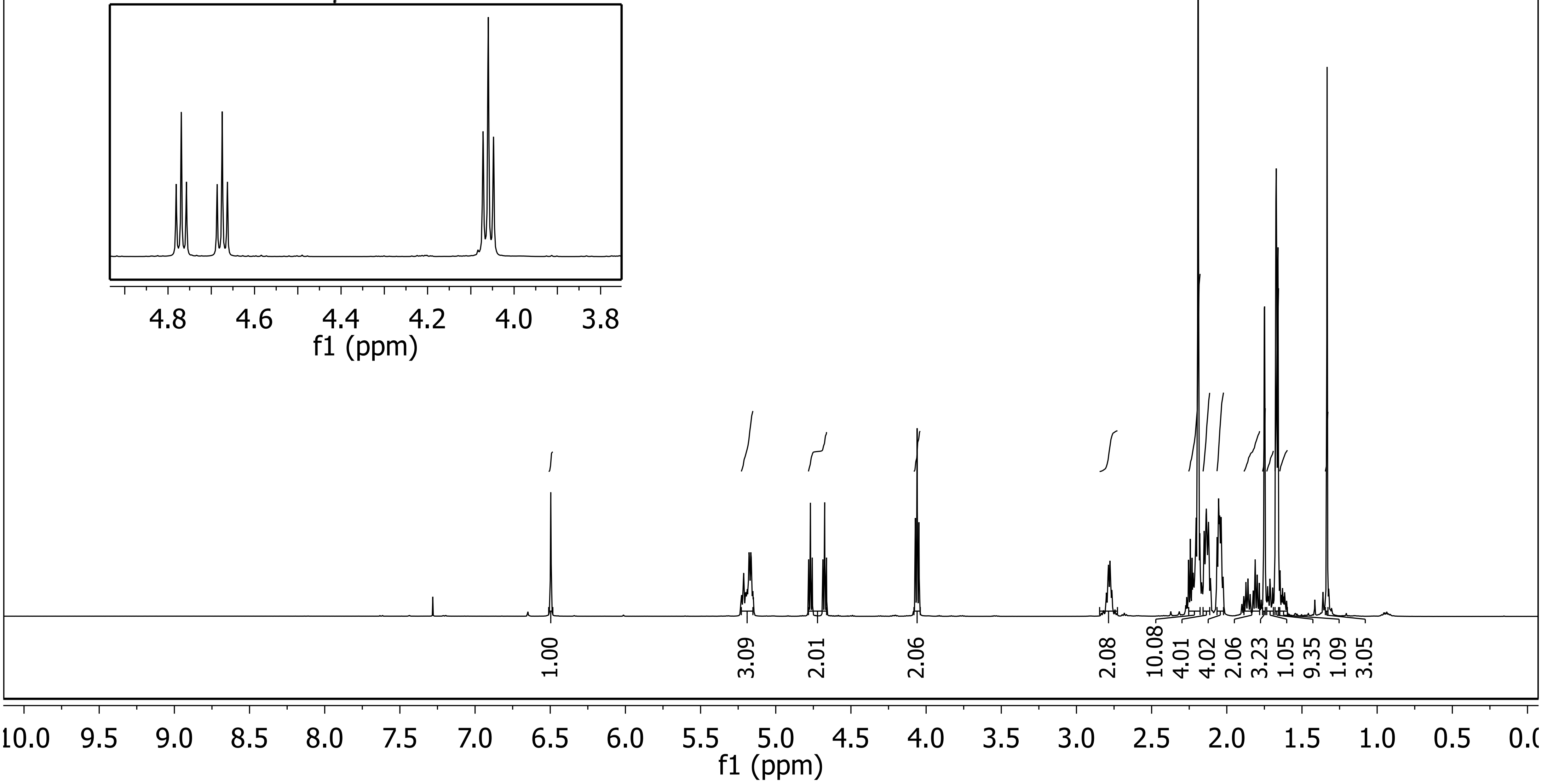

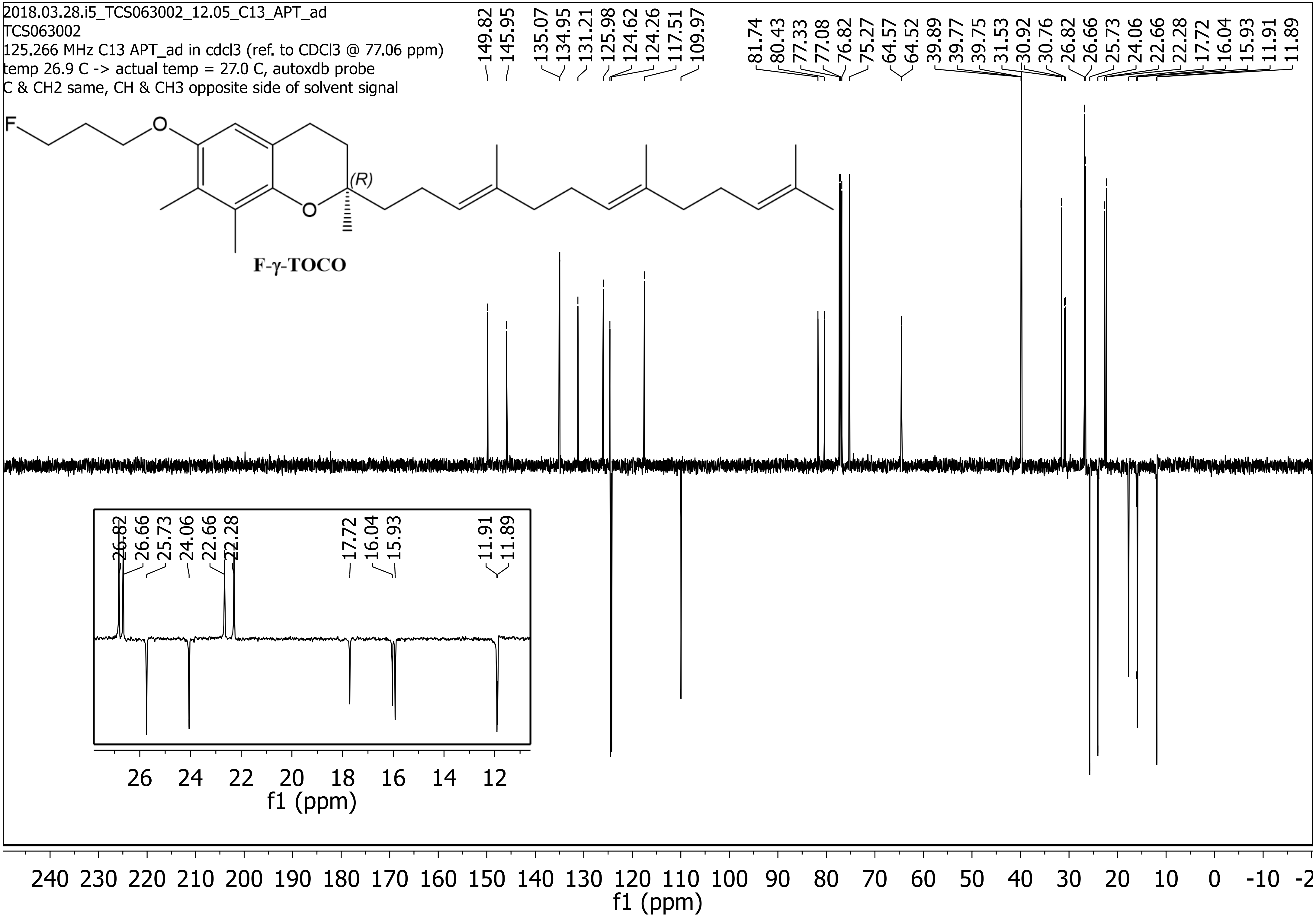
2018.03.28.i5_TCS063002_12.05_C13_APT_ad
TCS063002
125.266 MHz C13 APT_ad in cdcl3 (ref. to CDCl3 @ 77.06 ppm)
temp 26.9 C -> actual temp = 27.0 C, autoxdb probe
C & CH2 same, CH & CH3 opposite side of solvent signal
149.82
145.95
135.07
134.95
131.21
125.98
124.62
124.26
117.51
109.97
81.74
80.43
77.33
77.08
76.82
75.27
64.57
64.52
39.89
39.77
39.75
31.53
30.92
30.76
26.82
26.66
25.73
24.06
22.66
22.28
17.72
16.04
15.93
11.91
11.89
F
O
O
(R)
F-γ-TOCO
26 24 22 20 18 16 14 12
f1 (ppm)
240 230 220 210 200 190 180 170 160 150 140 130 120 110 100 90 80 70 60 50 40 30 20 10 0 -10 -2
f1 (ppm)

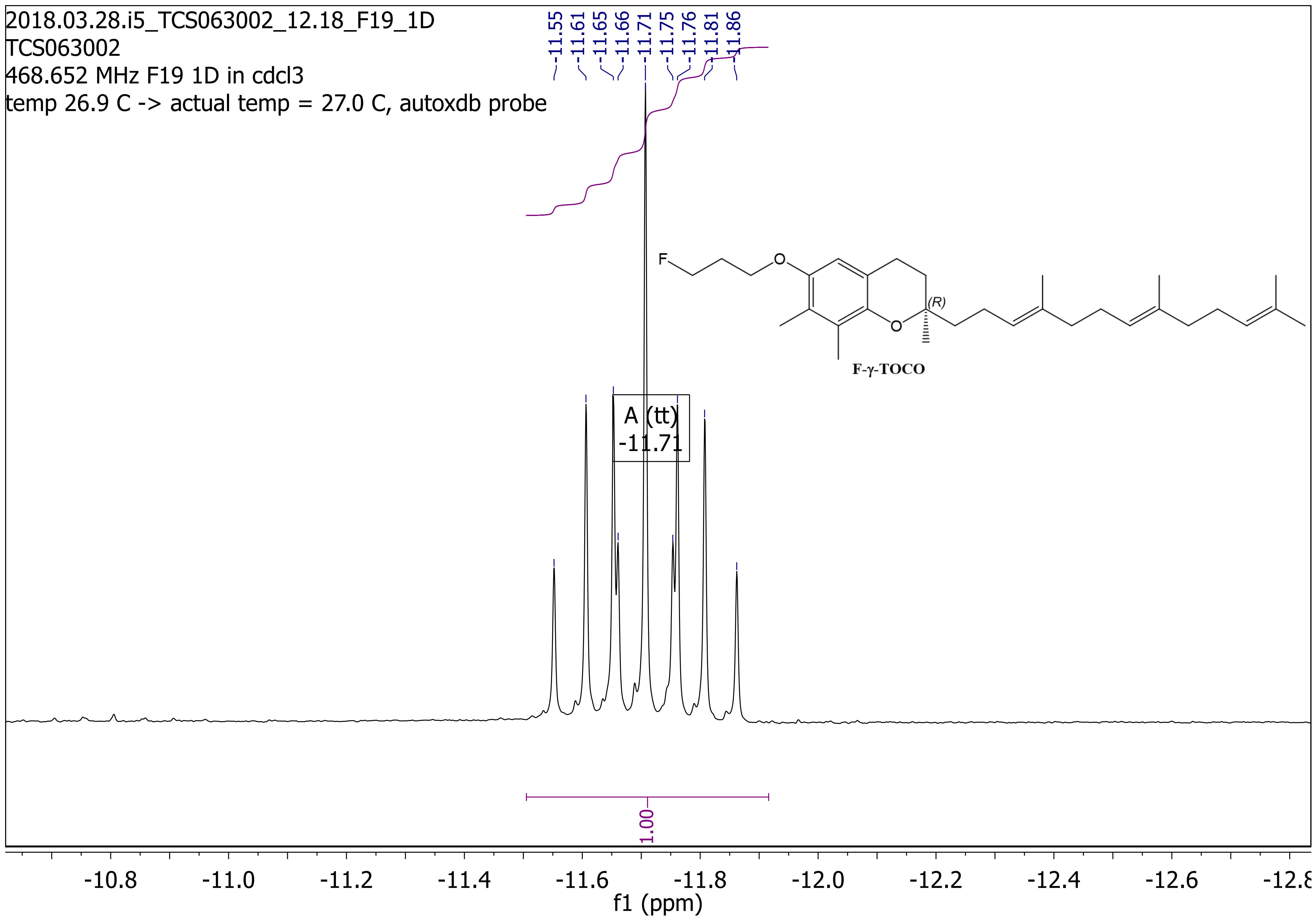
2018.03.28.i5_TCS063002_12.18_F19_1D
TCS063002
468.652 MHz F19 1D in cdcl3
temp 26.9 C -> actual temp = 27.0 C, autoxdb probe
-11.55
-11.61
-11.65
-11.66
-11.71
-11.75
-11.76
-11.81
-11.86
A (tt)
-11.71
F
O
O
(R)
F-γ-TOCO
1.00
-10.8
-11.0
-11.2
-11.4
-11.6
-11.8
-12.0
-12.2
-12.4
-12.6
f1 (ppm)

2018.05.11.i5_TCS063003_12.18_H1_1D
TCS063003
498.118 MHz H1 1D in cdcl3 (ref. to CDCl3 @ 7.26 ppm)
temp 26.9 C -> actual temp = 27.0 C, autoxdb probe

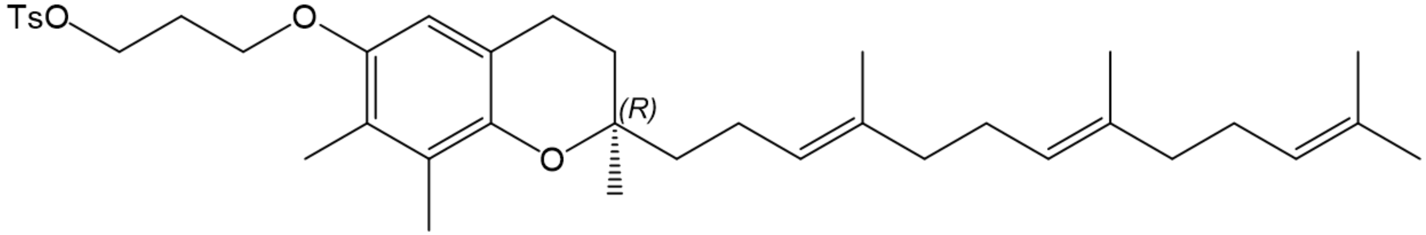

TsO-γ-TOCO

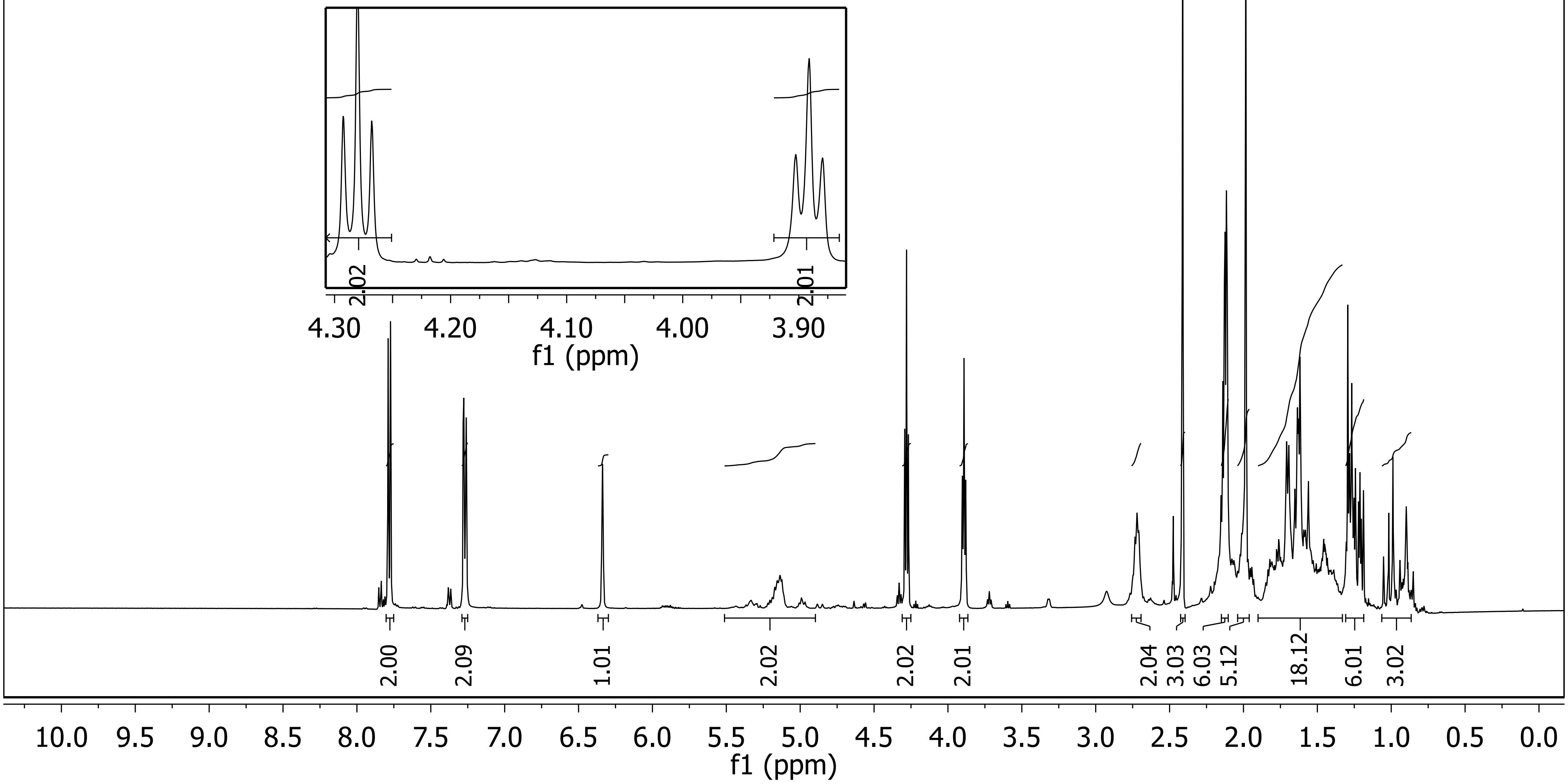

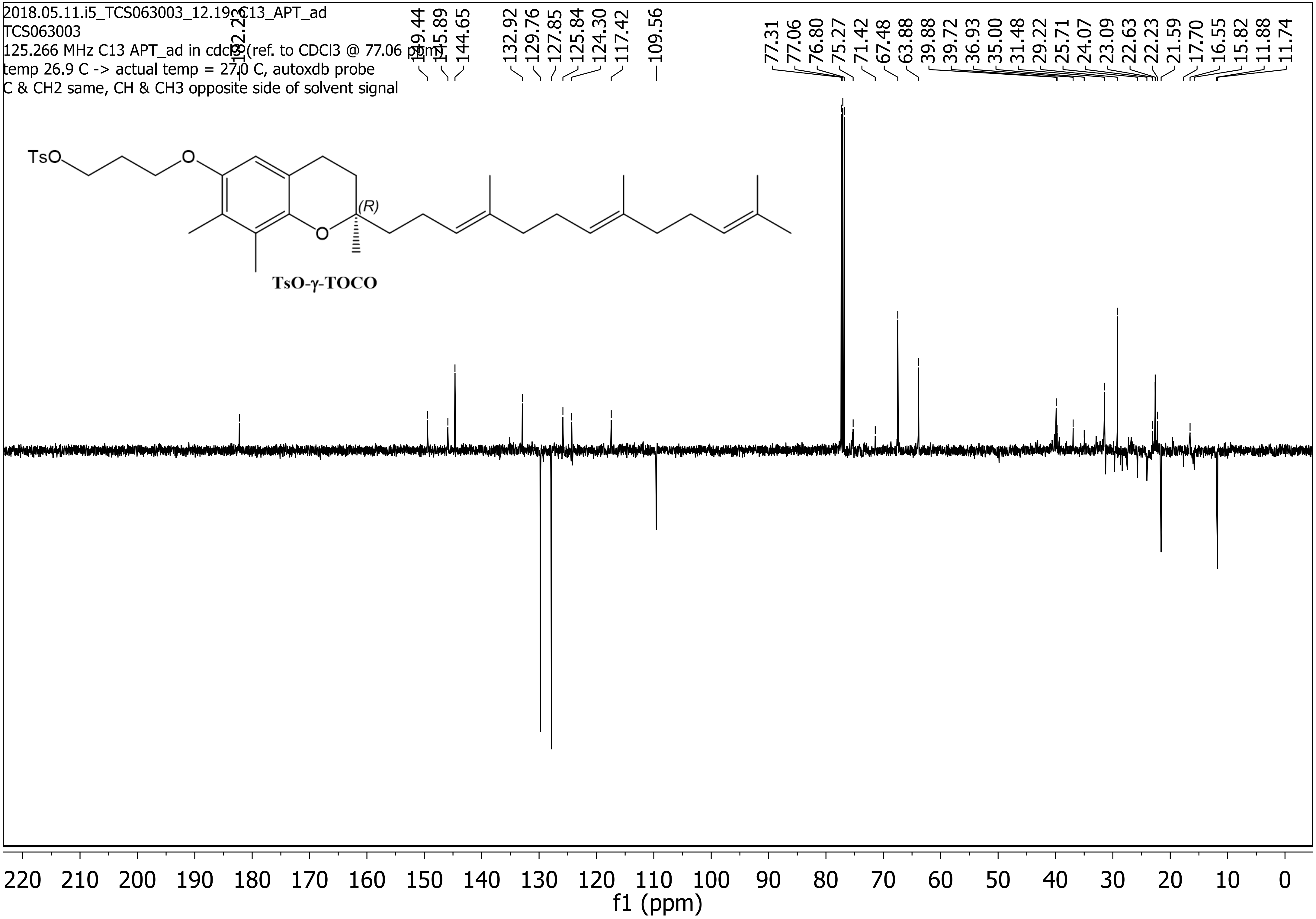
2018.05.11.i5_TCS063003_12.19.C13_APT_ad
TCS063003
125.266 MHz C13 APT_ad in cdcl3 (ref. to CDCl3 @ 77.06 ppm)
temp 26.9 C -> actual temp = 27.0 C, autoxdb probe
C & CH2 same, CH & CH3 opposite side of solvent signal
149.44
145.89
144.65
132.92
129.76
127.85
125.84
124.30
117.42
109.56
77.31
77.06
76.80
75.27
71.42
67.48
63.88
39.88
39.72
36.93
35.00
31.48
29.22
25.71
24.07
23.09
22.63
22.23
21.59
17.70
16.55
15.82
11.88
11.74
TsO
O
O
(R)
TsO-γ-TOCO
220 210 200 190 180 170 160 150 140 130 120 110 100 90 80 70 60 50 40 30 20 10 0
f1 (ppm)
